# Passage‐Dependent Size Distributions of Human‐Derived Cells: Implications for Metabolic Assays

**DOI:** 10.1096/fj.202600816RR

**Published:** 2026-06-10

**Authors:** Ermes Botte, Piera Mancini, Chiara Magliaro, Arti Ahluwalia

**Affiliations:** ^1^ Research Centre “E. Piaggio” University of Pisa Pisa Italy; ^2^ Department of Information Engineering University of Pisa Pisa Italy

**Keywords:** allometric scaling, biological variability, cell culture passages, cell size, size‐related functionality

## Abstract

Biological noise is ubiquitous in living systems; yet, it is often neglected in cell‐based experiments, potentially biasing data interpretation. We provide a quantitative characterization of single‐cell equivalent diameter distributions in six human cell types using cell counting. By analyzing thousands of cells over passage number, we show that cell size is phenotype dependent and varies significantly with passage, with most cell types exhibiting a progressive reduction in median diameter. This variability is structured: When diameters are converted to masses, the distributions obey scaling laws, revealing conserved statistical properties of human cells in culture. Because key physiological processes such as metabolism scale with cell mass, passage‐dependent shifts in diameter distributions can propagate into functional readouts. We show that changes in cell size are sufficient to bias estimates of construct‐level metabolic rate, potentially confounding the interpretation of size‐normalized assays and treatment effects. Our results highlight that biological noise in vitro is a source of statistical structure that enables scaling analyses and a dynamic property that, if ignored, can lead to systematic misinterpretation of experimental outcomes. Accounting for size distributions and their evolution over passages may therefore improve experimental design, data interpretation, and, ultimately, the translatability of in vitro models.

## Graphical abstract

Cell size changes with passage. This propagates into size‐dependent functional readouts, such as metabolic assays.

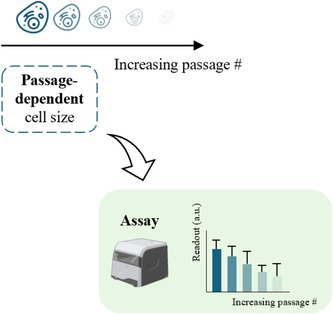

## 1 Introduction

Biological systems are well‐known to be stochastic, a pervasive feature whatever the level or scale under investigation (i.e., from molecular clusters up to populations of living organisms). This stochasticity is often referred to as *biological noise* and underlies the widely observed variability between individuals—be they cellular assemblies or ecological communities [1, 2, 3, 4, 5, 6].

Nevertheless, most biomedical studies on noise have focused on molecular fluctuations and gene regulation [7, 8, 9, 10], while the impact and significance of biological variability at higher scales—such as at the tissue, organ, system or population level—is typically neglected or reduced to standard dispersion indices (e.g., standard deviation). The lack of extensive data needed to properly characterize their fluctuations stems, at least in part, from the low throughput and high costs of experimental approaches traditionally exploited for measuring biological parameters at macroscales [11, 12]. Capturing the intrinsic variability of biological parameters is also challenging from a technical point of view, since the sensitivity of many techniques is often comparable to—and, therefore, hard to decouple from—the amplitude of their fluctuations, especially at the cellular scale [13]. Hence, inferences are generally based on averages. For instance, a plethora of excellent reports on metabolic measurements in humans (i.e., by respirometry) [14, 15], marine microorganisms (i.e., by quantification of the photosynthetic activity) [16, 17] or mammalian cells in vitro (i.e., by oxygen sensing) [18, 19] only discuss their datasets in terms of means and variances. Similarly, a number of studies on size‐related scaling limit their considerations to average values of physiological quantities involved (e.g., body size, anatomical dimensions, life history parameters, metabolic rates), overlooking how they distribute within the population(s) under study [15, 20, 21, 22, 23]. Such statistics represent an oversimplified description of phenotypic heterogeneities and could mask systematic effects with potential implications on emerging individual or collective dynamics; this might lead to misinterpretation of experimental data and potentially hinder the relevance and impact of studies.

However, during recent decades, the pivotal role of stochasticity in shaping individual behaviors and determining the susceptibility and response to external changes of living communities—spanning from cells in tissues and organs up to whole organisms in ecosystems—has been extensively highlighted [11, 12, 24, 25, 26, 27]. Characterizing their intrinsic variability is becoming a compelling need in life sciences, as we begin to understand it might be key for testing hypotheses, formulating more accurate mechanistic models, and probing the reliability of their predictions [11, 28, 29, 30].

In this context, as scaling relationships have empirically proven that size—be it expressed as characteristic length, volume or mass—constitutes one of the major determinants of several functional and behavioral traits of living systems across scales [31, 32, 33, 34, 35], describing size fluctuations could provide information on the variability of parameters which covary with it [11, 12, 36, 37]. Thus, measuring size variability in cell cultures constitutes a foundational step towards describing phenotypic fluctuations in in vitro systems. Moreover, integrating scaling laws with distribution statistics provides a seamless framework for the quantitative interpretation of biological fluctuations in a rigorous manner. Based on this rationale, we present a systematic characterization of human cell sizes in vitro for six different cell types. Single‐cell size was measured by means of equivalent diameter using automated cell counting in order to gather an easily accessible but extensive dataset. We were thus able to describe their intrinsic size variability in terms of probability distributions for each considered phenotype and evaluate key features, such as the dependency on passage number and scaling behavior, which may influence the interpretation of in vitro experiments.

The methods and approach proposed here could help lead to new experimental and design paradigms which enhance the predictivity and translatability of in vitro models through valorizing biological noise.

## 2 Materials and Methods

Cell sizes were measured for six phenotypes to characterize their probability distributions. We tested four human cancer cell lines and two non‐immortalized stem cell types from different providers, as specified in Table 1. The cell lines were chosen because they represent commonly used models for in vitro studies.

**TABLE 1:** Overview of cell types characterized in this study and number of cells measured per type.

Cell type	Description	Provider	Number of cells
HepG2	Human hepatocellular carcinoma cells	Cytion (Eppelheim, Germany)	13 047
A549	Human alveolar adenocarcinoma cells	ATCC (Manassas, Virginia, USA)	12 196
Caco‐2	Human colorectal adenocarcinoma cells	ATCC (Manassas, Virginia, USA)	3345
SH‐SY5Y	Human neuroblastoma cells from bone marrow	Merck (Darmstadt, Germany)	3066
ADSC	Adipose‐derived stem cells	ATCC (Manassas, Virginia, USA)	1824
NPC	Neural progenitor cells	Merck (Darmstadt, Germany)	5536

### 2.1 Cell Maintenance

Before each round of experiments, cells were cultured in T25 flasks (Sarstedt, Numbrecht, Germany) in an incubator (37°C, 5% CO_2_, 95% RH) according to the cell providers' guidelines (see Table 1). They were supplied with 5 mL of the appropriate fresh culture medium every 2–3 days (depending on the protocol) and expanded until 90% confluence was reached. At this stage, the medium was fully removed, and the cells were rinsed with 1.5 mL of phosphate buffered saline (PBS—Lonza, Basel, Switzerland). A volume of 1.5 mL of trypsin–EDTA (or equivalent prescribed proteolytic enzyme) was then added and left for 5 min in the incubator for cell detachment. Finally, an equal volume of medium was used to inactivate the enzyme, and residually adherent cells were collected by gently pipetting the suspension. Two thirds of the suspension was used to measure cell sizes.

### 2.2 Cell Size Measurements

The cell suspension was centrifuged for 5 min at 900 rpm (Centrifuge 5702, Eppendorf, Hamburg, Germany) and resuspended in 2 mL of fresh culture medium. A sample of 30 μL was then mixed with the same volume of Trypan Blue (Thermo Fisher Scientific, Walthan, Massachusetts, USA), and an automated imaging‐based cell counter (Countess II FL, Thermo Fisher Scientific) was used to determine cell number, viability and cell sizes in the sample. Specifically, cell size was quantified in terms of equivalent diameter (i.e., the diameter of a sphere with the same projected area as the cell), since it is known not to depend on isovolumetric changes of cell morphology [37] and thus represents a suitable metric for cell size. Note that the built‐in image processing algorithm of the cell counter allows discriminating single cells even within small clusters, therefore avoiding potential artifacts due to residual cell aggregates after trypsinization. At least 3 samples per day were characterized using this procedure. The cell suspension was incubated in a 15 mL tube (Sarstedt) between consecutive samplings, and only viable cells according to the Trypan Blue test were considered for the characterization. As detailed in the Supporting Information, analyses of intra‐day repeatability and inter‐day reproducibility confirmed the precision of measurements and excluded artifacts arising from instrumental drift over time.

### 2.3 Data Processing

**FIGURE 1:**
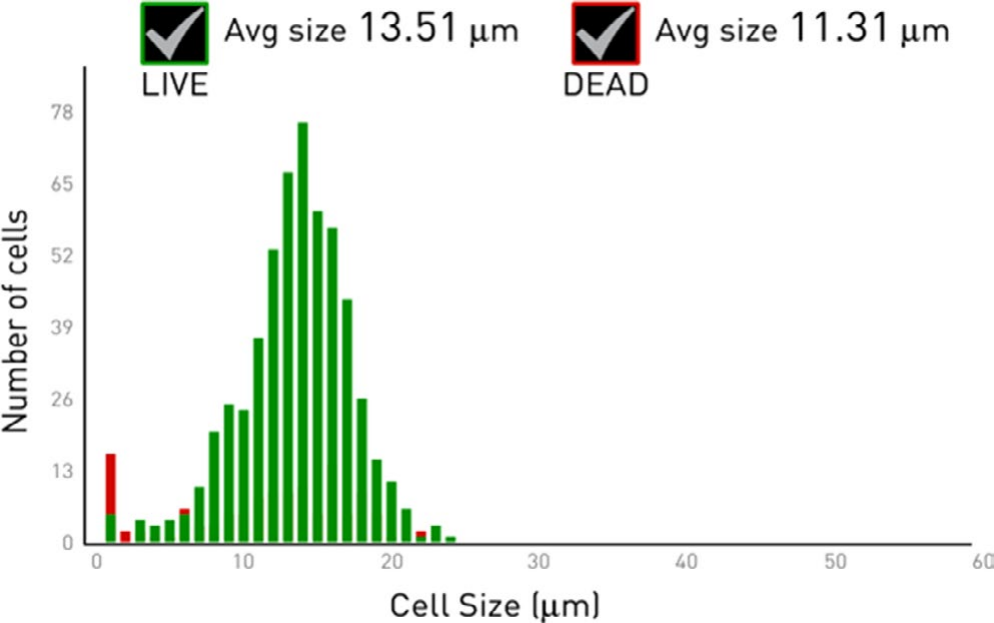
Example of the equivalent cell diameter distribution as provided by the cell counter. The histogram image and average values of cell size for both live (green) and dead (red) cells are reported.

For each readout, the cell counter provides the distribution of equivalent diameters estimated for all the cells detected in the sample in the form of the associated histogram, distinguishing between live and dead cells (Figure 1). The output for live cells was pre‐processed in Matlab (R2022b, The Mathworks, California, USA) to remove outliers and exclude any aggregates overlooked by the cell counter. Readouts from the cell counter were then integrated into a cumulative dataset describing relative frequency distributions of cell size, as well as arrange such raw data for statistical analysis.

### 2.4 Statistical and Scaling Analysis

The frequency distributions obtained were first tested for normality by means of the Lilliefors test and characterized in terms of descriptive statistics using Matlab (R2022b, The Mathworks, California, USA). Then, the non‐parametric Kruskal‐Wallis test was applied in GraphPad Prism (v9, GraphPad Software, California, USA) to infer statistical differences among size medians of different cell types as well as of samples of the same cell type measured after different passages. GraphPad Prism was also used to perform correlation and regression analyses.

The computational framework previously developed by our group for scaling analyses of mass‐related biological parameters [12] was then used to assess whether single‐cell mass distributions derived from equivalent diameters measured here display scaling properties, as already reported elsewhere for dry masses of unicellular organisms [38]. To perform this analysis, equivalent cell diameters ($d_{\mathrm{eq}}$) were converted to masses (m) as follows:

(1) $$m=\frac{\pi }{6}\omega {d_{\mathrm{eq}}}^{3}$$

assuming that the density of cells ω is equal to that of water (1000 kg m^−3^). Then, the inter‐phenotype scaling exponent of cell masses with respect to their average value was determined by applying a procedure technically referred to as “collapse”. It consists in identifying the exponent value corresponding to the maximal overlap of frequency distributions, after proper normalization according to the theory reported in references [11, 12, 38] (refer to the Supporting Information for further analytical details) [11, 12, 38]. Finally, the Anderson‐Darling test allows comparing shapes of such normalized distributions and thus evaluating whether they significantly collapse onto each other.

## 3 Results

### 3.1 Characterization of Cell Size Distributions

**FIGURE 2:**
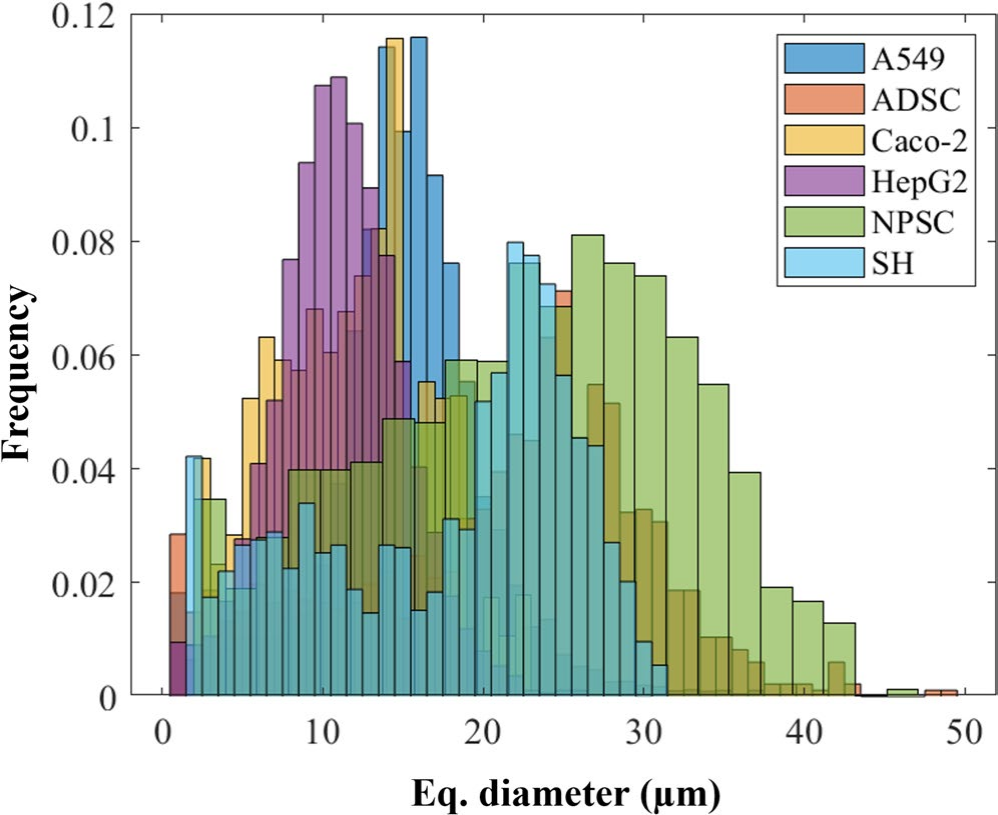
Relative frequency distributions of single‐cell sizes or equivalent diameters measured for six human phenotypes.

Figure 2 shows frequency distributions of equivalent cell diameters measured for each phenotype. The Lilliefors test (α = 0.05) proved that the distributions are neither sampled from normal nor lognormal probability density functions (*p* < 0.001), therefore non‐parametric statistics were computed for all populations. The position (median value), dispersion (inter‐quartile range or IQR) and shape index (skewness) of the diameter distribution for each cell type are reported in Table 2.

**TABLE 2:** Descriptive parameters of equivalent diameter distribution for the 6 cell types investigated.

Cell type	Median (μm)	IQR (μm)	Skewness
HepG2	11	5	0.19
A549	15	5	−0.07
Caco‐2	11	8	0.10
SH‐SY5Y	20	14	−0.46
ADSCs	23	13	−0.40
NPCs	24	15	−0.23

### 3.2 Inter‐ and Intra‐Phenotype Comparisons

**FIGURE 3:**
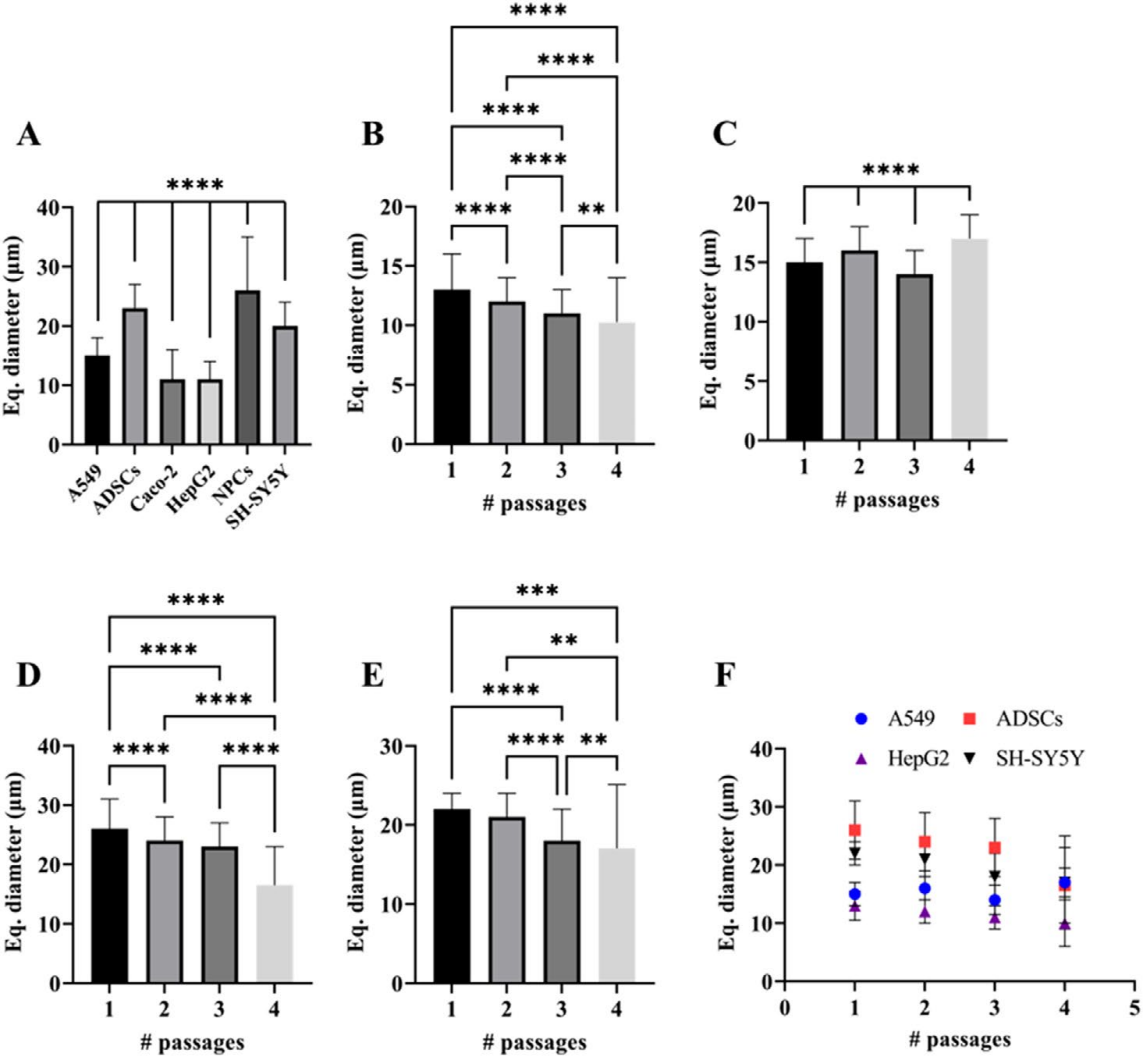
Inter‐ and intra‐phenotype comparisons, indicating that human cell size in vitro depends on both the cell type and the number of passages. (A) Equivalent diameters measured for six human phenotypes (Kruskal‐Wallis test, *p* < 0.0001—see Table 2 for sample sizes). Equivalent diameters measured over 4 subsequent passages for (B) HepG2 cells, (C) A549 cells, (D) ADSCs and (E) SH‐SY5Y cells (Kruskal‐Wallis test, *p* < 0.0001 in all cases). A statistically significant decrease across passages emerges in (B, D and E, F). Summary of the observed trends of cellular equivalent diameters over passage number. All datasets are expressed as median ± IQR. Keys for Dunn's post hoc comparisons: **p < 0.005, ****p < 0.0001.

Statistically significant differences among median diameters of different cell types were identified using the Kruskal‐Wallis test (α = 0.05, *p* < 0.0001, Figure 3A). Cell sizes measured over 4 subsequent passages are reported in Figure 3B–E for HepG2 cells, A549 cells, ADSCs and SH‐SY5Y cells, respectively. Again, the Kruskal‐Wallis test (α = 0.05) highlighted that statistically significant changes in terms of median diameter occur across passages for all 4 cell types (*p* < 0.0001), an aspect which is typically overlooked when performing cell culture experiments. More specifically, a decreasing trend of equivalent diameter over passage number is observed in the case of HepG2, ADSCs and SH‐SY5Y (see Figure 3B,D,E), suggesting that cells cultured in monolayers may gradually shrink as a function of the number of detachment‐plating cycles they undergo. The scatter plot in Figure 3F summarizes these emerging passage‐dependent trends of median diameters for the tested phenotypes. Importantly, note that—from Figure 3B–F—the horizontal axes do not refer to the absolute number of passages for that line but correspond to the progressive sequence of passages in which we measured cell size.

**FIGURE 4:**
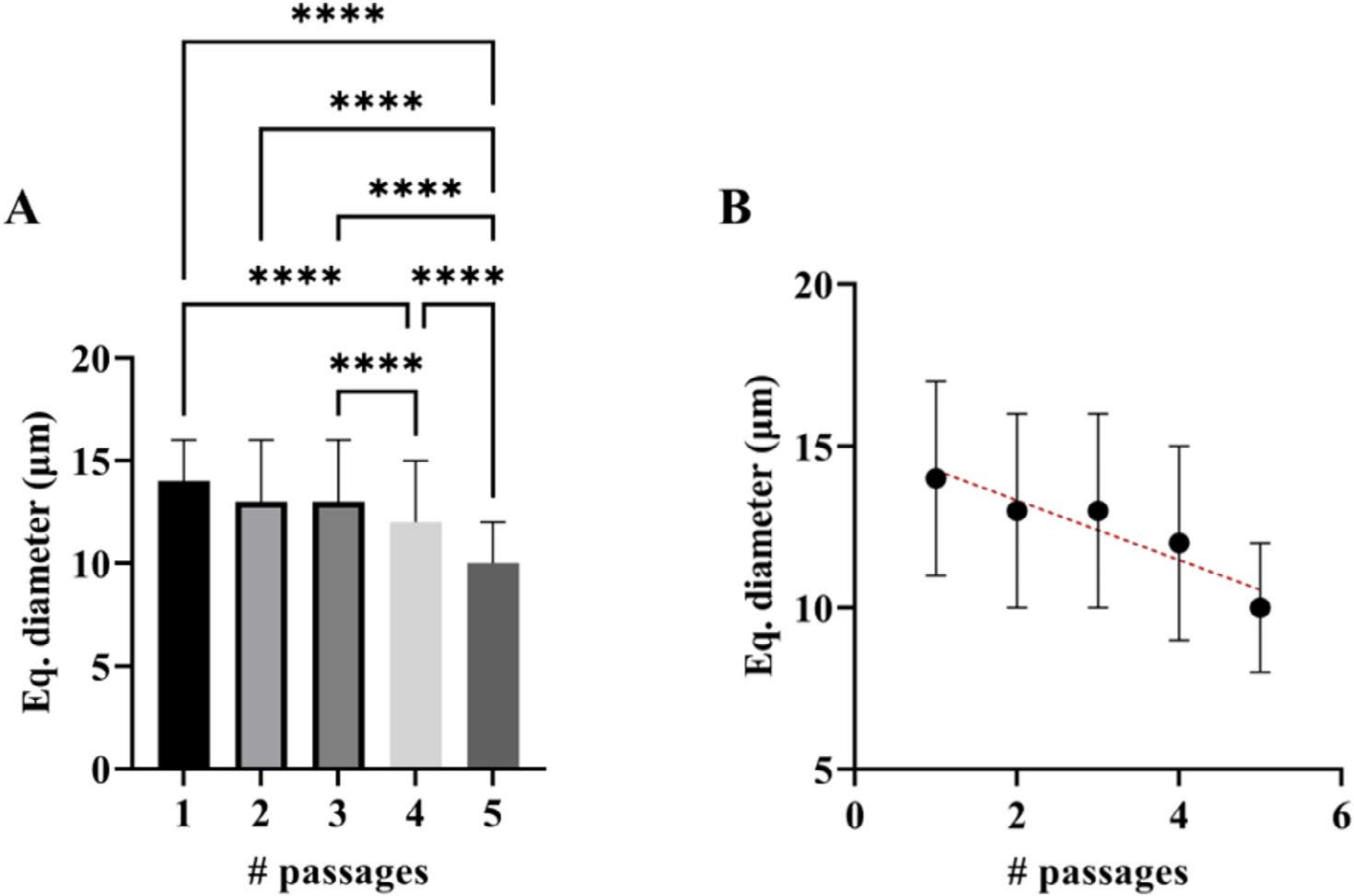
Intra‐phenotype comparison for HepG2 cells. (A) Equivalent diameters measured over 5 subsequent passages are different from each other (Kruskal‐Wallis test, *p* < 0.0001). (B) Linear trend of decreasing equivalent diameters over passage number (*R* ^*2*^ = 0.9026, the slope of the regression line differs from zero with *p* = 0.0133). All datasets are expressed as median ± IQR. Keys for Dunn's *post hoc* comparisons: ****p < 0.0001.

An analysis of this intra‐phenotype dynamics over a more extended observation window of 5 subsequent passages is depicted in Figure 4 for HepG2 cells. The Kruskal‐Wallis test (α = 0.05) confirmed that their size varies statistically between passages (*p* < 0.0001, Figure 4A). In particular, the equivalent diameter linearly correlates with the number of passages (Spearman correlation coefficient *r* = −0.9747, with *p* = 0.0333), displaying a decreasing trend (*R* ^2^ = 0.9026, Figure 4B) with slope significantly different from zero (*p* = 0.0133).

A non‐parametric power analysis was performed to assess the adequacy of sample sizes for the statistical comparisons across passages. In all cases, the number of cells characterized per passage was sufficient to achieve a statistical power greater than 0.8, supporting the robustness of the reported inferences (see Table S1 in the Supporting Information).

### 3.3 Inter‐Phenotype Scaling of Cell Size Distributions

**FIGURE 5:**
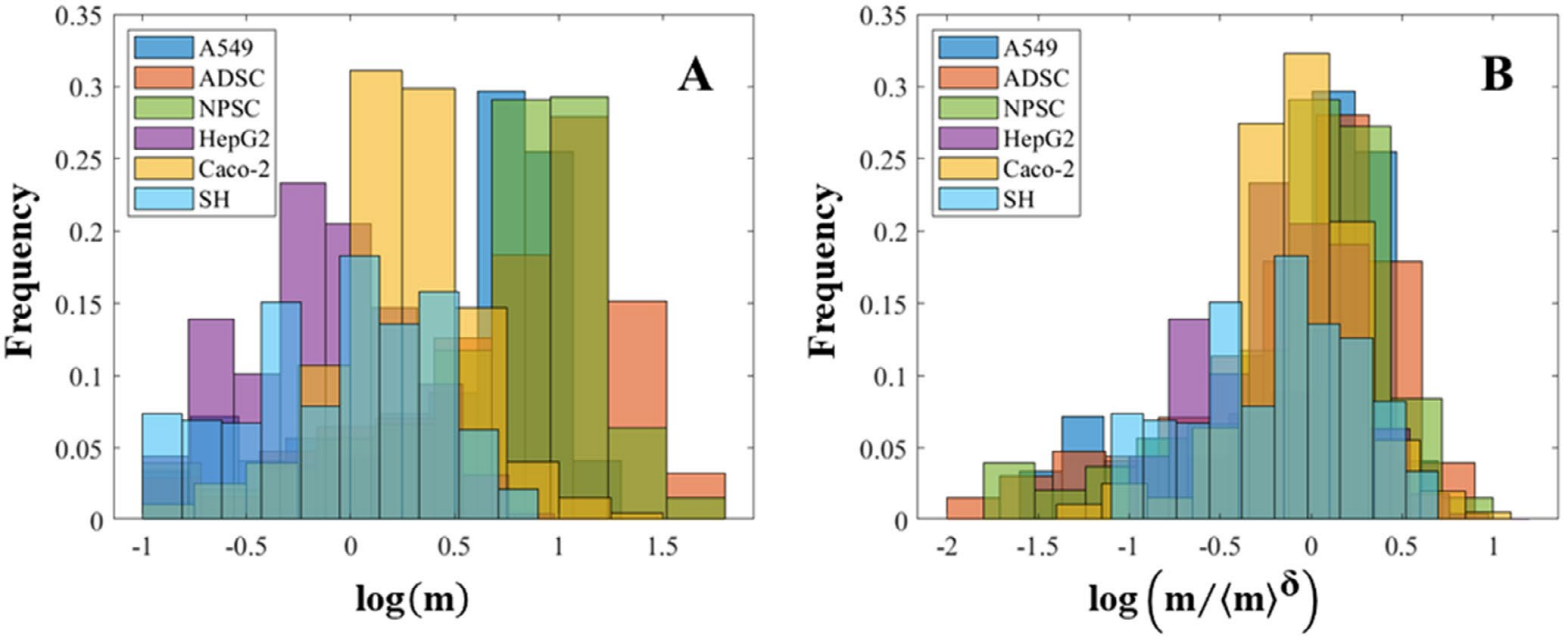
Inter‐phenotype scaling of human single‐cell mass distributions derived from measured equivalent diameters (using Equations (1) and (2)). (A) Relative frequency distributions before scaling. (B) Collapsed relative frequency distributions after scaling with δ = 0.87 ± 0.17.

Figure 5 reports the results of the scaling analysis, showing single‐cell mass distributions before (Figure 5A) and after (Figure 5B) data collapse. Remarkably, human single‐cell mass distributions exhibit a scaling exponent δ = 0.87 ± 0.17, with rescaled distributions collapsing onto a common scaling function (Anderson‐Darling test; α = 0.01, *p* = 0.0112). According to the criterion adopted in Botte et al. [12], δ is not statistically distinguishable from 1 as the identified range partially overlaps with 1.00 ± 0.10, and therefore isometric scaling cannot be excluded. A similar inter‐phenotype scaling behavior was previously reported for unicellular organisms by Giometto et al. [38] but has not been shown for human cells. At the same time, the uncertainty associated with the estimated exponent also overlaps with δ = 0.75, indicating that quarter‐power scaling [34] cannot be ruled out.

### 3.4 Propagation of Passage‐Dependent Size Changes Into Metabolic Rate Estimates

Given the well‐known relationship between mass (*m*) and metabolic rate (*B*) through allometric scaling (*B* ∝ *m* ^*δ*^) [39], we estimated the extent of metabolic variation of cell‐laden constructs having predefined size and cell density as a function of the passage‐dependent decrease in single‐cell equivalent diameters. Specifically, introducing ${∆}_{\%}d_{\mathrm{eq}}$ as the relative change of single‐cell size over a given passage window ∆t (i.e., ${∆}_{\%}x=\left(x\left(t+∆t\right)-x\left(t\right)\right)/x\left(t\right)$), we derived the corresponding relative variation of the whole‐construct basal metabolic rate (B, expressed in oxygen mol s^−1^) as:

(2) $${∆}_{\%}B=3\delta {∆}_{\%}d_{\mathrm{eq}}$$

where δ is the scaling exponent relating the mass of single cells to their maximal oxygen consumption rate [40]. The analytical details for deriving Equation (2) are reported as Supporting Information. Although it relies on certain assumptions (i.e., perfect spherical shape, uniform cell density, homogeneous oxygen consumption rate per cell), the equation underlines the functional consequences of the observed trend of cell size over passages (Figures 3 and 4). For example, even when metabolic assays are normalized to cell number, passage‐dependent changes in single‐cell size can alter the per‐cell metabolic contribution, potentially biasing the interpretation of functional readouts.

## 4 Discussion

Fluctuations are ubiquitous in nature and influence the structure, function, and behavior of living systems across scales. In cell‐based research, however, phenotypic variability is often treated as experimental noise and reduced to average values. As many physiological processes—at both cellular and organismal scales—are known to depend on cell size [33, 40], we characterized variability through a quantitative analysis of single‐cell size distributions across six human cell types over multiple passages.

Using widely accessible automated imaging‐based cell counting techniques—which provide a direct geometric estimate of cell size—we reconstructed probability distributions of equivalent cell diameters from large cell populations; complementary methods such as flow cytometry yield indirect proxies (e.g., forward scatter) and require calibration for quantitative interpretation. The analysis shows that cell size, quantified here through the equivalent diameter, is both phenotype dependent and passage dependent. Different cell types display distinct median diameters, and, within the same phenotype, median size shifts significantly over subsequent passages. In particular, for HepG2 cells we observed a progressive reduction in median diameter over five passages which follows a linear trend.

These results indicate that cell size is not an inherent or fixed property of a given phenotype, but rather an evolving characteristic influenced by culture history. Moreover, they highlight that biological variability in cell populations is structured rather than purely random, even when captured through simple geometric descriptors.

When equivalent diameters are converted to cell mass, the resulting mass distributions exhibit conserved scaling behavior, consistent with previous observations in unicellular organisms [11, 12, 38]. Although the uncertainty associated with estimated exponent does not allow discriminating between isometric or non‐isometric scaling, the collapse of distributions reveals a shared statistical structure. Albeit based on the assumption that cells are approximately spherical, this result supports the applicability of scaling frameworks that incorporate biological variability to human cells cultured in vitro.

Importantly, the same statistical structure that enables scaling analyses also implies that shifts in measured size distributions can propagate into mass‐dependent functional readouts. Because metabolic rate scales with cell mass, passage‐dependent reductions in single‐cell diameter are expected to alter metabolic demand at both the single‐cell and construct level, even when cell number and culture geometry are held constant. Thus, passage‐induced size drift represents a potential source of bias in functional assays.

This has practical implications for the interpretation of metabolic measurements commonly used in vitro. Resazurin‐based assays (e.g., Alamar Blue), for instance, are widely employed as proxies for cell viability or activity and are typically normalized to cell number, assuming that each cell contributes equally to the measured signal. However, if per‐cell metabolic activity depends on cell mass, then passage‐dependent reductions in size may modify the reducing capacity per cell. Consequently, differences in resazurin readouts observed across passages or experimental conditions may partly reflect underlying shifts in size distributions rather than intrinsic treatment effects, suggesting that normalizing by the total cellular mass rather than the cell number may be more meaningful. These considerations are particularly relevant in three‐dimensional cultures, where metabolic assays are often used to infer construct‐level functionality.

More broadly, these findings suggest that cell size should be considered when designing experiments and scheduling culture routines. A key strength of the present approach is that it relies on widely available automated cell counting, enabling high‐throughput quantification of single‐cell size distributions from large populations without requiring specialized instrumentation. This allowed us to analyze multiple phenotypes and passages with sufficient statistical power to support the reported inferences. Comparisons across wide passage windows or between datasets with unmatched passage histories—which are often the case because of practical timings and low throughput of measurement techniques—may introduce hidden biases in size‐ or mass‐normalized assays. Monitoring and reporting of cell size distributions may therefore help disentangle genuine biological responses from culture history‐dependent drifts.

Finally, by demonstrating that cell mass inferred from size measurements in human cells follows scaling behavior, this study provides a framework for linking size distributions to mass‐dependent biological functions, such as metabolism. Quantitative datasets that integrate variability with functional readouts remain scarce in life sciences [11, 37], yet they are essential to move beyond average‐based descriptions and to understand how variability propagates across scales. Incorporating structured biological variability into in vitro analysis may therefore strengthen the design and evaluation of tissue models, where mass‐dependent metabolic scaling has been proposed as a criterion for assessing translational value [12, 41, 42].

## Author Contributions

A.A., C.M. and E.B. conceived the research. E.B. and P.M. performed experimental measurements, developed the software, and processed the collected datasets. E.B. ran statistical analysis, contributed analytical tools, and drafted the manuscript. All the authors interpreted results and reviewed the manuscript before submission.

## Funding

The work leading to these results has been funded by the Italian Ministry of University and Research in the framework of Fondo Italiano per la Scienza, through the project FIS‐2024 DYNAMITE (CUP: I53C25002130001).

## Conflicts of Interest

The authors declare no conflicts of interest.

## Supporting information


**Table S1:** Smallest sample size per passage (*n*), achieved statistical power (*pwr*) and minimum sample size per passage required to ensure statistical power of 0.8 (*n*_*min*_) for non‐parametric hypothesis testing (Figure 3 in the main text) and linear regression analysis (Figure 4 in the main text).
**Figure S1:** Overlapping relative frequency distributions of equivalent diameters measured in three different replicates during the same day. (A) HepG2, (B) A459 and (C) SH‐SY5Y cells. No statistically significant differences were observed (Kruskal‐Wallis test, p > 0.0776), confirming the intra‐day repeatability of single‐cell size measurements.
**Figure S2:** Overlapping relative frequency distributions of equivalent diameters measured on different days in HepG2 cells at the same passage. (A) Passage 6, (B) passage 7 and (C) passage 8. No statistically significant differences were detected (Mann–Whitney test, p > 0.1190), confirming the inter‐day repeatability of single‐cell size measurements.

## Data Availability

All data collected in the study are available in open‐access repository (https://zenodo.org/records/19671582?token=eyJhbGciOiJIUzUxMiJ9.eyJpZCI6IjU3MWU2N2Y2LWFlNDctNGMwZS04OGIwLTQzNThjNTI0MWJmYSIsImRhdGEiOnt9LCJyYW5kb20iOiIzZWFlYjMyYTczMjUwZTczMjJkNTdiYjExYjk3MzIxYyJ9.enquTZMw1LNyDEHRU5VVAX0KVyVj7SOWD‐‐WhJw8R1324‐grOCrqt5obkU_OXYzI3h2i9cXjayYC9x2rUj4jXA).
