# Supplementary material for: Passage‐Dependent Size Distributions of Human‐Derived Cells: Implications for Metabolic Assays
Source: FASEB J. 2026 Jun 10;40(12):e71993. doi: 10.1096/fj.202600816RR (PMC13252009; doi:10.1096/fj.202600816RR)
Supplement: Supplementary file 1 — Table S1: Smallest sample size per passage (n), achieved statistical power (pwr) and minimum sample size per passage required to ensure statistical power of 0.8 (n min ) for non‐parametric hypothesis testing (Figure 3 in the main text) and linear regression analysis (Figure 4 in the main text). Figure S1: Overlapping relative frequency distributions of equivalent diameters measured in three different replicates during the same day. (A) HepG2, (B) A459 and (C) SH‐SY5Y cells. No statistically significant differences were observed (Kruskal‐Wallis test, p > 0.0776), confirming the intra‐day repeatability of single‐cell size measurements. Figure S2: Overlapping relative frequency distributions of equivalent diameters measured on different days in HepG2 cells at the same passage. (A) Passage 6, (B) passage 7 and (C) passage 8. No statistically significant differences were detected (Mann–Whitney test, p > 0.1190), confirming the inter‐day repeatability of single‐cell size measurements. [file FSB2-40-e71993-s001.docx]

Supplemental Information

Passage-dependent size distributions of human-derived cells: implications for metabolic assays

Ermes Botte^a,b,+^, Piera Mancini^a,b,+^, Chiara Magliaro^a,b^ and Arti Ahluwalia^a,b,^*

*^a^* Research Centre “E. Piaggio”, University of Pisa

Largo L. Lazzarino 1, 56122 Pisa, Italy

*^b^* Department of Information Engineering, University of Pisa

Via G. Caruso 16, 56122 Pisa, Italy

^*^ corresponding author, e-mail address: [arti.ahluwalia@unipi.it](mailto:arti.ahluwalia@unipi.it)

^+^ these authors equally contributed to the work

**1. Supplemental Information Text**

**1.1 Assessment of intra- and inter-day reproducibility**

To assess the repeatability of single-cell size measurements obtained by automated cell counting across consecutive runs, we first compared triplicates of equivalent diameter distributions acquired on the same day for three different cell types (namely, HepG2, A549 and SH-SY5Y cells – see **Figure S1**). No significant differences were detected among replicates (Kruskal-Wallis test, *p* > 0.0776).

We also evaluated inter-day reproducibility by repeating the characterization protocol on different days for HepG2 cells at the same passage number (passages 6, 7 and 8 – **Figure S2**). Again, no statistically significant differences were observed between equivalent diameter distributions (Mann-Whitney test, *p* > 0.1190), indicating that instrumental drift was negligible over the time window considered. Together, these results support the precision and stability of the methodology, within the limits of intrinsic biological fluctuations of cell size.

The statistical analyses were performed using GraphPad Prism.

**1.2 Power analysis**

Power analysis was performed in Matlab’s Statistics and Machine Learning Toolbox. **Table S1** compares the (smallest) size of collected samples per passage (*n*) to the minimum required to guarantee an adequate statistical power of 0.8 (*n_min_*), as well as the power we actually achieved using at least *n* measurements per passage (*pwr*). Even the smallest samples are greater than the estimated minimum size per passage, supporting the robustness of the reported inferences (subsection 3.2 in the main text).

**Table S1.** Smallest sample size per passage (n), achieved statistical power (pwr) and minimum sample size per passage required to ensure statistical power of 0.8 (n_min_) for non-parametric hypothesis testing (Figure 3 in the main text) and linear regression analysis (Figure 4 in the main text).

| **Cell type** | **Analysis** | ***n*** | ***pwr*** | ***n_min_*** |
| --- | --- | --- | --- | --- |
| HepG2 | Kruskal-Wallis | 3270 | 0.95 | 2859 |
| A549 | Kruskal-Wallis | 745 | > 0.99 | 354 |
| SH-SY5Y | Kruskal-Wallis | 424 | 0.96 | 325 |
| ADSCs | Kruskal-Wallis | 303 | 0.97 | 184 |
| HepG2 | Linear regression | 511 | > 0.99 | 20 |

**1.3 Introducing variability in allometric scaling analyses**

According to the theory first proposed by Giometto *et al*. and Zaoli *et al*.,^[1,2]^ single-cell mass distributions can be described by the general probability density function in Equation (S1):

| $p\left( m \vert\left\langle m \right\rangle\right)= \frac{1}{m}F\left( \frac{m}{\left\langle m \right\rangle^{\delta}} \right)$ | (S1) |
| --- | --- |

where $p\left( m | \left\langle m \right\rangle\right)$ is the probability distribution of cell masses conditioned to its average value $\left\langle m \right\rangle$, while $F\left( \frac{m}{\left\langle m \right\rangle^{\delta}} \right)$ denotes a scaling function common to the whole set of distributions. If so, leveraging their self-similarity, such distributions are assumed to collapse onto each other when normalized by $\left\langle m \right\rangle^{\delta}$. Therefore, the value of $\delta$ corresponding to the best possible collapse is a descriptor of the scaling behaviour accounting for biological fluctuations.

**1.4 Single-cell size changes imply metabolic rate shifts**

Here we report on analytical details for assessing whether and how the dependency on the passage number of single-cell size (quantified by means of equivalent diameter, $d_{eq}$) impacts on the overall metabolic rate ($B$) of cell-laden spheroids.

Given the central role of oxygen (O_2_) in the majority of biochemical processes,^[3]^ *B* is intended as a descriptor of O_2_ metabolism, *i.e.*, the number of O_2_ moles consumed per unit time at steady state (in mol s^-1^). In stationary conditions, the diffusion of O_2_ within the construct balances its consumption and can thus be described by the reaction-diffusion equation as in Equation (S2):

| $- \boldsymbol{\nabla}\cdot\boldsymbol{J}\left( r,t\to\infty\right) {- \varphi}_{O2}=0$ | (S2) |
| --- | --- |

where $\boldsymbol{\nabla}= \left( \begin{matrix} \frac{\partial}{\partial r} \\ \frac{1}{r}\frac{\partial}{\partial\theta} \\ \frac{1}{r*sin\theta}\frac{\partial}{\partial\varphi} \end{matrix} \right)$ is the nabla operator in spherical coordinates, $\boldsymbol{J}$ (mol m^-2^ s^-1^) is the O_2_ flux field, and $\varphi_{O2}$ (mol m^-3^ s^-1^) stems for the overall consumption rate. Integrating Equation (S1) over the volume of the spheroid (having radius *R*) and considering that $\iiint\boldsymbol{\nabla}\cdot\boldsymbol{J}\left( r,t\to\infty\right)dV= ∯ \boldsymbol{J}\left( R,t\to\infty\right)\cdot d\boldsymbol{S}$ according to Gauss’ theorem, we obtain Equation (S3):

| $∯ \boldsymbol{J}\left( R,t\to\infty\right)\cdot d\boldsymbol{S}= \iiint\varphi_{O2}dV$ | (S3) |
| --- | --- |

where both sides are in mol s^-1^ and represent equivalent definitions.

Assuming homogeneous cell density ($\rho_{cell}$, in cells m^-3^) and zero-order uptake kinetics (*i.e.*, that $\varphi_{O2}$ is independent of the O_2_ concentration and constant in space and time), we can build on Equation (S3) to write Equation (S4):

| $B= \frac{4}{3}\pi R^{3}\varphi_{O2}= \frac{4}{3}\pi R^{3}\rho_{cell}sOCR$ | (S4) |
| --- | --- |

with *sOCR* (mol cell^-1^ s^-1^) the average O_2_ consumption rate of a single cell within the cell-laden construct. Leveraging the universality of size-related scaling,^[4]^ *sOCR* can be expressed as a function of single-cell size according to Equation (S5):

| $sOCR=am^{\delta}=a\left( \frac{\pi}{6}\omega{d_{eq}}^{3} \right)^{\delta}$ | (S5) |
| --- | --- |

where *m* is the single-cell mass, $\omega$ = 1000 kg m^-3^ is the density of cells (equal to that of water), *a* is a normalization constant, and $\delta$ indicates a generic scaling exponent. Then, putting Equations (S4) and (S5) together, *B* can be re-written as follows:

| $B=\frac{4}{3}\pi R^{3}\rho_{cell}a\left( \frac{\pi}{6}\omega\right)^{\delta}{d_{eq}}^{3\delta}= \Gamma{d_{eq}}^{3\delta}$ | (S6) |
| --- | --- |

where $\Gamma$ incorporates the construct-related constants. Equation (S6) implies a power-law relationship between the O_2_ metabolism of the whole spheroid (*B*) and the size distribution of cells ($d_{eq}$) it contains.

Considering the observed shrinking trend over passages (Figures 2 and 3 in the main text), we can estimate the change of *B* over a passage window $\Delta t$ as $\Delta B=B\left( t+\Delta t \right)-B\left( t \right)$. $\Delta t$ corresponds to a cell size variation $\Delta d_{eq}$ (with $\Delta d_{eq}<0$, as cell size decreases). In the limit of $\Delta d_{eq} \to0$ (and, hence, $\Delta t \to0$), one can calculate the derivative in Equation (S7).

| $\frac{\partial B}{\partial d_{eq}}=3\delta\Gamma{d_{eq}\left( t \right)}^{3\delta-1}= \frac{3\delta}{d_{eq}\left( t \right)}B\left( t \right)$ | (S7) |
| --- | --- |

Separating variables and resorting back to finite differences (that is $\frac{\partial B}{\partial d_{eq}} \approx\frac{\Delta B}{\Delta d_{eq}}$), we obtain Equation (S8).

| $\frac{\Delta B}{B\left( t \right)}= 3\delta\frac{\Delta d_{eq}}{d_{eq}\left( t \right)}$ | (S8) |
| --- | --- |

Finally, introducing the relative variations $\Delta_{\%}d_{eq}= \frac{\Delta d_{eq}}{d_{eq}\left( t \right)}$ and $\Delta_{\%}B= \frac{\Delta B}{B\left( t \right)}$, we get:

| $\Delta_{\%}B=3\delta\Delta_{\%}d_{eq}$ | (S9) |
| --- | --- |

as Equation (3) in the main text**.**

**2. Supplemental Information Figures**


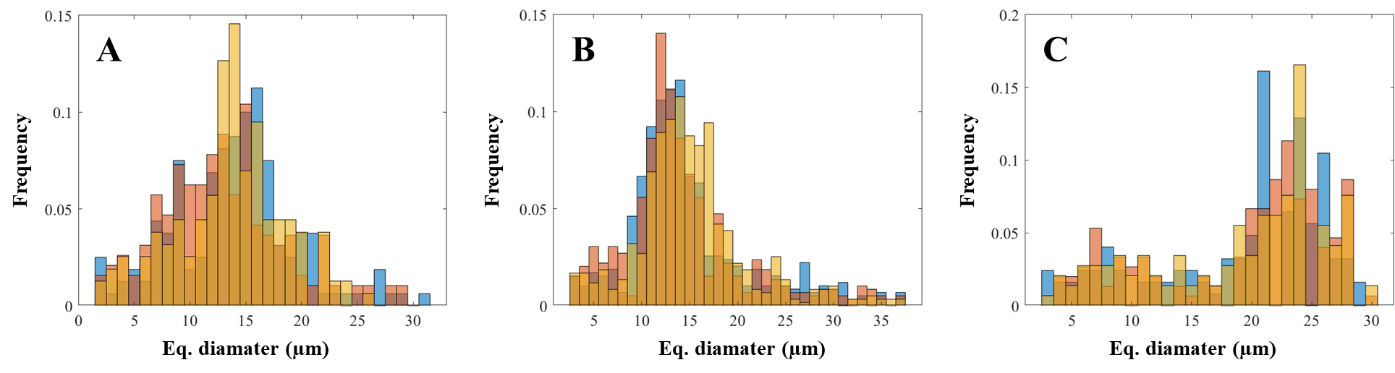


**Figure S1**. Overlapping relative frequency distributions of equivalent diameters measured in three different replicates during the same day. **A**) HepG2, **B**) A459 and **C**) SH-SY5Y cells. No statistically significant differences were observed (Kruskal-Wallis test, p > 0.0776), confirming the intra-day repeatability of single-cell size measurements.


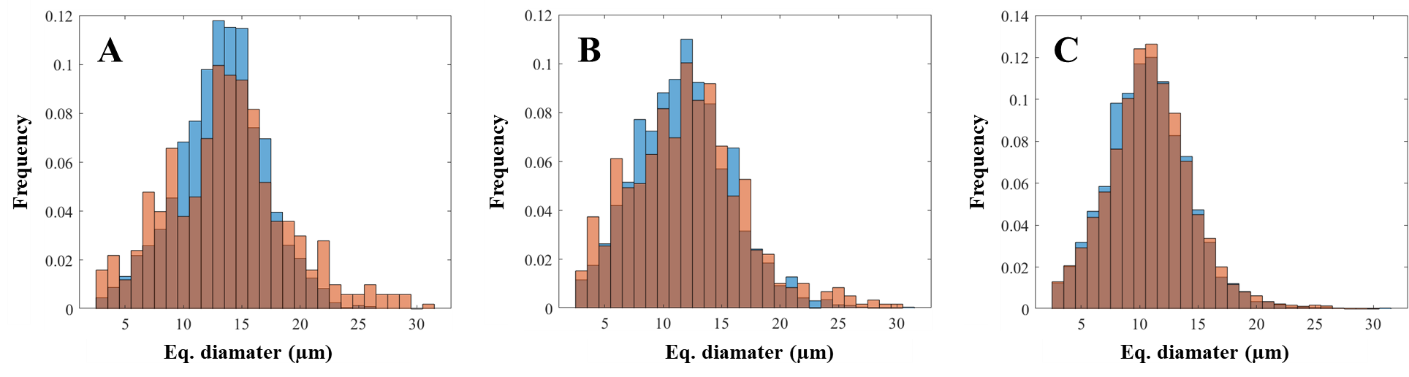


**Figure S2**. Overlapping relative frequency distributions of equivalent diameters measured on different days in HepG2 cells at the same passage. **A**) Passage 6, **B**) passage 7 and **C**) passage 8. No statistically significant differences were detected (Mann-Whitney test, p > 0.1190), confirming the inter-day repeatability of single-cell size measurements.

**3. References**

[1] A. Giometto, F. Altermatt, F. Carrara, A. Maritan, A. Rinaldo, *Proc. Natl. Acad. Sci. U. S. A.* **2013**, *110*, 4646.

[2] S. Zaoli, A. Giometto, E. Marañón, S. Escrig, A. Meibom, A. Ahluwalia, R. Stocker, A. Maritan, A. Rinaldo, *Proc. Natl. Acad. Sci. U. S. A.* **2019**, *116*, 17323.

[3] S. Rose, *The Chemistry of Life*, Penguin Books, **1999**.

[4] G. B. West, W. H. Woodruff, J. H. Brown, *Proc. Natl. Acad. Sci. U. S. A.* **2002**, *99*, 2473.
